# Acetabular labral tear complicating idiopathic osteonecrosis of the femoral head treated by labral repair with hip arthroscopy: a case report

**DOI:** 10.1186/1752-1947-8-372

**Published:** 2014-11-18

**Authors:** Hiroyuki Izumida, Arihiko Kanaji, Toru Nishiwaki, Hidenori Shimizu, Atsuhiro Fujie, Toshimi Tando, Yoshiaki Toyama, Yasunori Suda

**Affiliations:** 1 Department of Orthopedic Surgery, School of Medicine, Keio University, 35 Shinanomachi, Shinjuku, Tokyo 160-8582, Japan

**Keywords:** Hip arthroscopy, Idiopathic osteonecrosis, Labral tear

## Abstract

**Introduction:**

It has been well documented that labral tear is frequently associated with femoroacetabular impingement and dysplasia of the hip; however, there have been few reported cases of labral tear associated with idiopathic osteonecrosis of the hip. Here we report the case of a patient with labral tear associated with idiopathic osteonecrosis of the femoral head who was treated by hip arthroscopy, with a favorable short-term outcome.

**Case presentation:**

Under the diagnosis of systemic lupus erythematosus, a 28-year-old Japanese woman was treated with the oral administration of steroid in 2007. A year after the treatment, she developed right hip joint pain and was diagnosed with idiopathic osteonecrosis of the femoral head at our institution. In November of 2011, she revisited our hospital when her right hip joint pain exacerbated and she became unable to walk. On the visit, the anterior impingement sign and Patrick test were positive. Radiography and magnetic resonance imaging in 2011 demonstrated neither spreading of the osteonecrosis area nor collapse of the femoral head in the right joint; however, magnetic resonance imaging showed a high-intensity area in the articular labrum in a T2-weighted image, leading to a diagnosis of labral tear. She underwent labral repair with hip arthroscopy in August of 2012. Now, 1 year after surgery, she does not feel any pain during walking and her modified Harris hip score has improved from 20 prior to surgery to 85.

**Conclusion:**

The case indicated that it is important to be aware of the possibility of labral tear in patients with idiopathic osteonecrosis of the femoral head, when spreading of the osteonecrosis area or collapse of the femoral head has not been seen on magnetic resonance imaging.

## Introduction

Favorable outcomes of labral tear treated using hip arthroscopy have recently been reported, and the indication of hip arthroscopy has been expanding. However, the cause of complaints of patients with idiopathic osteonecrosis (ION) of the femoral head may involve deformity and destruction of the hip joint accompanying osteonecrosis, and there has been no report focusing on labral lesions.

We applied arthroscopic hip surgery to a patient with labral tear complicating ION of the femoral head, and achieved a favorable short-term outcome.

## Case presentation

A 28-year-old Japanese woman was diagnosed with Sjögren syndrome and systemic lupus erythematosus at the Department of Dermatology of our hospital in 2006, and oral treatment with 20mg of prednisolone was initiated. She felt right coxalgia with no inducer in 2007, and was diagnosed with ION of the right femoral head at our department, but the pain was transient and remitted. Periodic follow-up was continued thereafter, but pain in her right hip joint over the gluteal region started upon sitting for a prolonged time in November 2011. Because the pain gradually aggravated and she became unable to walk, she revisited our department in August of 2012.

**Figure 1:**
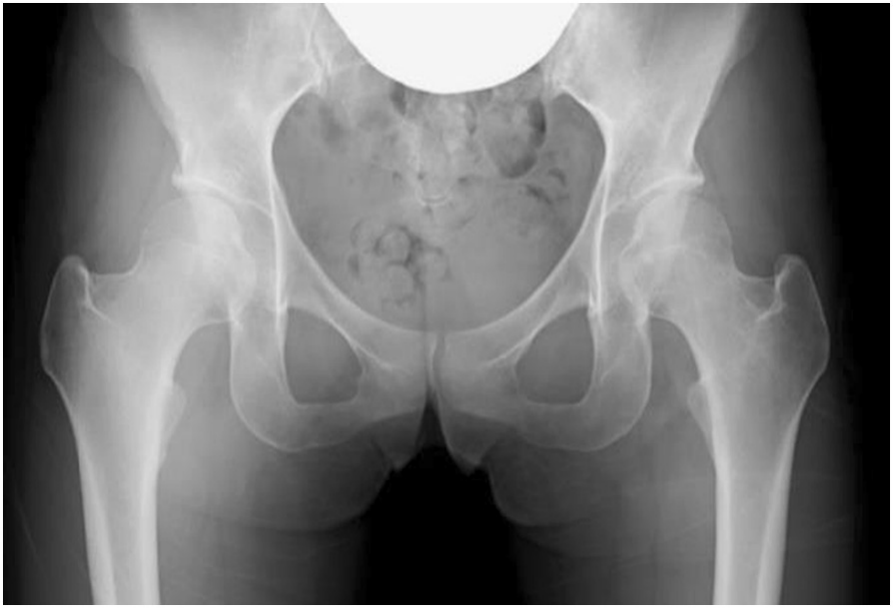
**Plain radiogram of the hip joints on revisit.** The cross-over sign was noted on the right side. The center-edge and sharp angles were 29° and 54°, respectively, showing mild dysplasia of the hip joint.

**Figure 2:**
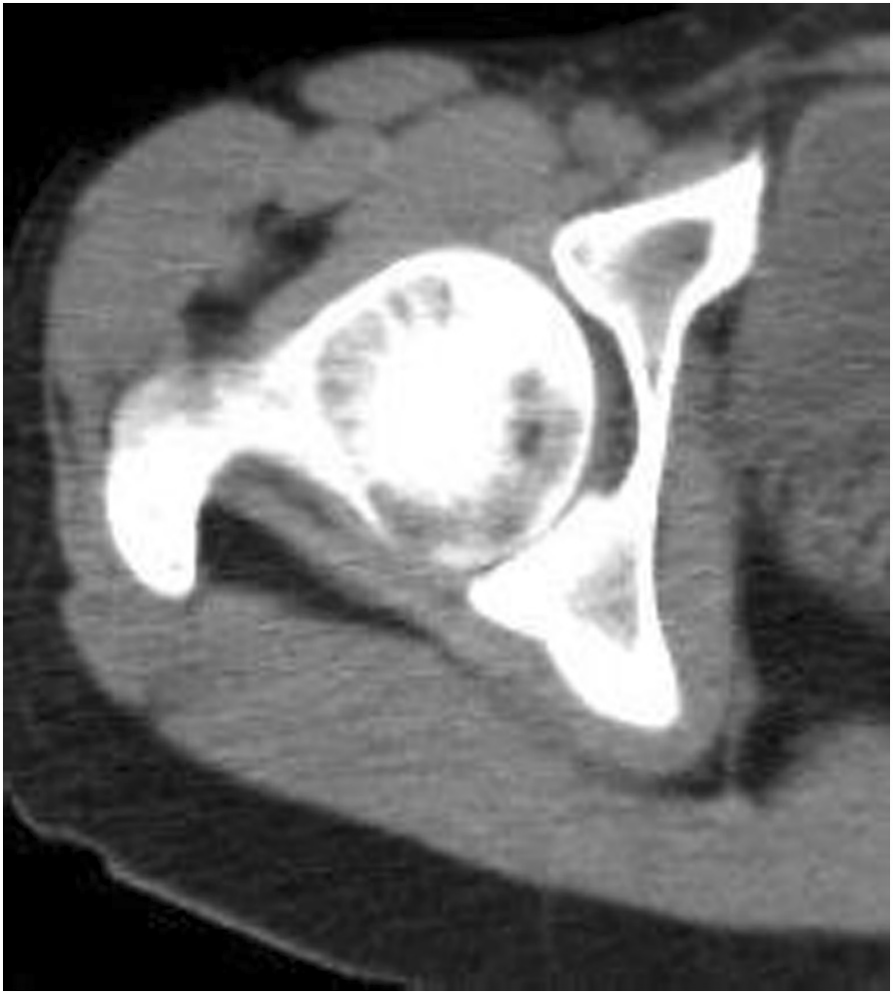
Computed tomography: the α angle was 50°, and no cam lesion was observed.

**Figure 3:**
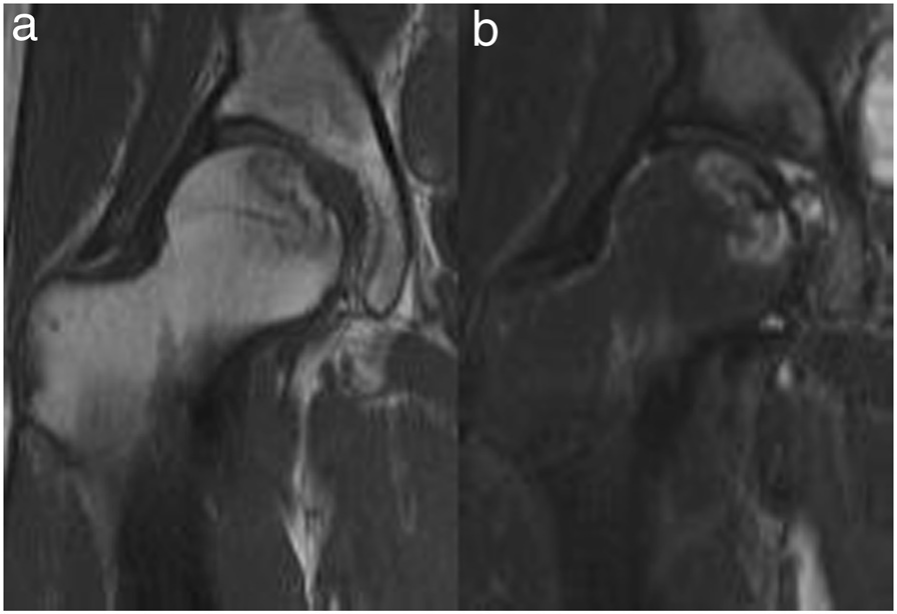
**Preoperative magnetic resonance imaging. a**. T1-weighted imaging: a band sign characteristic of idiopathic osteonecrosis of the femoral head was observed. **b**. T2 short-tau inversion recovery: a high intensity was noted in the acetabular labrum, but no bone marrow edema or hydrarthrosis suggesting collapse was present in the necrotic region of the femoral head.

At her revisit to our department, she felt right coxalgia accompanied by dysbasia, and antalgic claudication was noted. Mild tenderness was noted in the Scarpa triangle, and internal rotation was slightly limited in the range of motion of her right hip joint. The anterior impingement sign was positive but the Patrick sign was negative. Based on the modified Harris hip score (HHS), the score of pain on her right side was 10, and the total score was 20. Plain radiography of her bilateral hip joints demonstrated no collapse of the femoral head. The cross-over sign was positive, and the center-edge (CE) and sharp angles were 29° and 54°, respectively, showing mild acetabular dysplasia and pincer femoroacetabular impingement (FAI; Figure 1). Computed tomography of her hip joint showed that the α angle was 50°, and an osseous change suggesting a cam lesion was not noted in the femoral neck. No irregular joint surface was noted (Figure 2). T1-weighted imaging of magnetic resonance imaging (MRI) showed the band sign, which is the characteristic MRI finding of ION of the femoral head. The MRI findings also demonstrated that the patient had type B of the femoral head according to the Nishii *et al.* classification and stage 1 of the femoral head according to the Association Research Circulation Osseous classification [1,2]. On MRI T2 short-tau inversion recovery a high intensity was noted in the acetabular labrum, but no bone marrow edema or hydrarthrosis suggesting collapse was present in the necrotic region of the femoral head (Figure 3). A xylocaine test was performed on admission. When 3mL of xylocaine was injected into her right hip joint under fluoroscopy, right coxalgia was alleviated but not completely resolved on the following day, and long-distance walking was difficult due to residual pain while walking.

**Figure 4:**
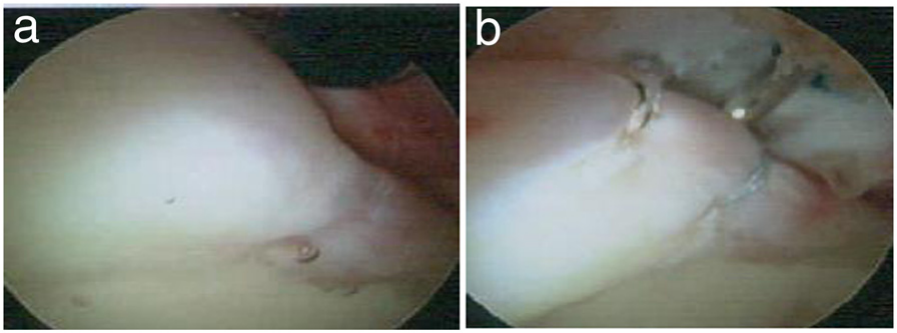
**Intraoperative findings. a** Before the labral suture. **b** Just after the labral suture.

**Figure 5:**
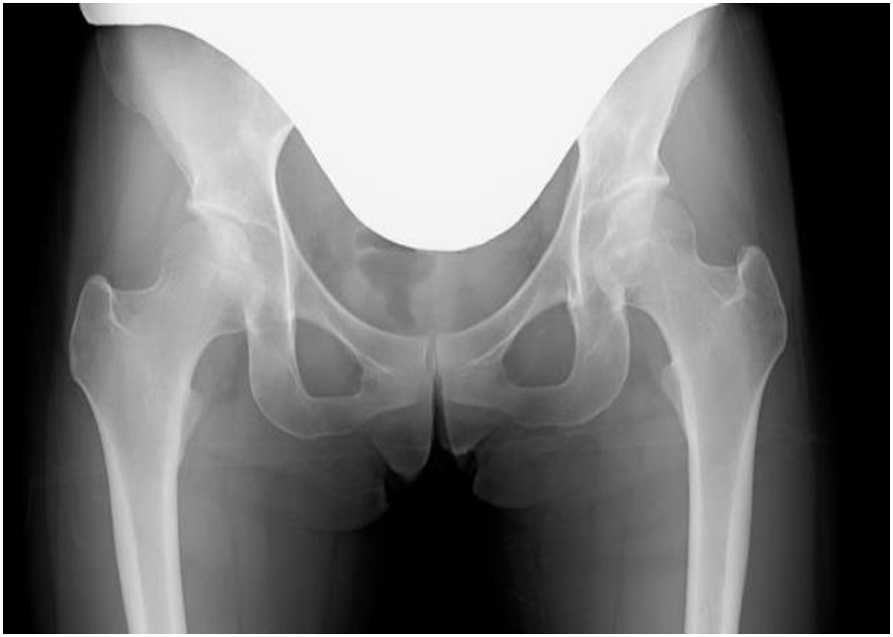
**Plain radiograph of the bilateral hip joints after surgery.** No narrowing of the joint space or collapse of the femoral head was noted.

Based on the findings that severe coxalgia-inducing dysbasia had developed, but the range of necrosis of the femoral head was narrow, inducing no collapse, the anterior impingement sign was positive, the xylocaine test was positive, and a finding suggesting acetabular labral tear was noted on MRI, the patient was diagnosed with labral tear complicating ION of the femoral head. Although mild acetabular dysplasia was present, because the CE angle was greater than 20° and tear and instability of the acetabular labrum were noted on arthroscopy, we performed labral repair with hip arthroscopy in September 2012.With the patient in a supine position, her lower leg was distracted using an operating table with a distraction system. An anterolateral portal was prepared under fluoroscopy, and arthroscopy was initiated. The anterior labrum was not only sagged like a hammock but also accompanied by cartilage delamination, showing the features of labral tear. A mid-anterior portal was prepared without transecting the articular capsule, and two stiches of suture were applied to the acetabular labrum using an anchor suture. No treatment for a pincer or cam lesion was applied. After suture, the fixability of the acetabular labrum was evaluated using a probe, and it was favorable without abnormal mobility. After sufficient irrigation, the skin incision was subcutaneously sutured and surgery was completed (Figure 4).Active exercise of her right joint started on the day following surgery, and 0.5 and full weight bearing were permitted at 2 and 3 weeks after surgery, respectively. No pain while walking was noted on the final follow-up at 1 year after surgery; she has resumed normal daily activities. No narrowing of the joint space or collapse of the femoral head was noted on plain radiography of her bilateral hip joints at the final follow-up (Figure 5), and the modified HHS had improved to 85 from 20 before surgery.

## Discussion

FAI and acetabular dysplasia have been reported to be causes of labral tear, and these are associated with deformity of bony elements of the hip joint [3-5]. Necrosis of the femoral head is a totally different disease from these, and it is unclear whether or not it causes labral tear. In this patient, no collapse of the femoral head, which is the cause of complaints of osteonecrosis of the femoral head, arthritis induced by the primary disease, bone marrow edema, or secondary osteoarthritis was noted, and it was difficult to explain the cause of severe coxalgia-inducing dysbasia. Based on the clinical findings that the anterior impingement sign and xylocaine test were positive and MRI findings, we suspected the complication of a labral tear, rather than searching for a direct cause in ION of the femoral head. When pain is noted despite no finding suggesting collapse using various imaging methods, such as observed in this patient, it may be necessary to consider the presence of another causative disease on examination. In this case, preoperative plain radiography demonstrated that the cross-over sign was positive, and the CE and sharp angles were 29° and 54°, respectively, suggesting that the labral tear of this patient was mainly induced by the acetabular dysplasia and pincer FAI.

It is controversial whether labral tear should be treated by partial resection or suture. The acetabular labrum has three functions: suction to stabilize the joint by maintaining negative pressure in the joint, sealing to promote a constant intra-articular pressure distribution, and increasing the joint cartilage area and acetabular volume. Schilders *et al*. and Larson *et al.* reported that the outcome of treating labral tear with suture was more favorable than resection of the acetabular labrum because suture improves sealing close to the normal state [6-10]. Suture was applied in our patient, and the modified HHS improved to 85 from 20 before surgery, although the duration of follow-up was short (1 year). In particular, pain was markedly improved and the course has been favorable, but acetabular dysplasia and FAI, which might have been the cause, were not treated, for which course observation should be continued in consideration of re-tear.

The progression of necrosis and possibility of collapse of the femoral head in the future should also be considered in the course observation. Regarding the natural course of osteonecrosis of the femoral head, since Sugano *et al.* reported in 1994 that the collapse rates of types A, B, and C were 0, 0, and 75%, respectively, in a study following 16 joints for 5 years on average, various researchers consider that the risk of collapse of the femoral head increases as the area ratio of the necrotic region increases in the weight-bearing region [11,12]. However, Nishii *et al.* reported that the collapse rates of types A, B, and C were 24, 50, and 76% in a study following 54 joints for 6 years on average, suggesting that collapse occurs even in types A and B [1]. We are planning to periodically perform close examination of the patient for not only re-tear of the labrum but also collapse of the femoral head using MRI, and consider the application of rotational osteotomy of the femoral head when the development of collapse of the femoral head is suspected.

## Conclusions

We encountered a patient with labral tear complicating osteonecrosis of the femoral head. Labral tear may occur in patients with painful osteonecrosis of the femoral head, to which attention should be paid in examination.

## Consent

Written informed consent was obtained from the patient for publication of this case report and any accompanying images. A copy of the written consent is available for review by the Editor-in-Chief of this journal.

## Abbreviations

CE: Center-edge; FAI: Femoroacetabular impingement; HHS: Harris hip score; ION: Idiopathic osteonecrosis; MRI: Magnetic resonance imaging.

## Competing interests

The authors declare that they have no competing interests.

## Authors’ contributions

HI wrote this manuscript. AK is the corresponding author, and supervised the manuscript. TN, HS, AF, TT, YT and YS participated in the patients’ therapy and helped to draft the manuscript. All authors read and approved the final manuscript.
